# A worm’s life: AMPK links muscle mitochondrial dynamics to physical fitness and healthy aging in *Caenorhabditis elegans*

**DOI:** 10.20517/jca.2023.14

**Published:** 2023-05-04

**Authors:** Vihang A. Narkar

**Affiliations:** Center for Metabolic and Degenerative Diseases, Brown Foundation Institute of Molecular Medicine, UTHealth McGovern Medical School, Houston, TX 77030, USA.

**Keywords:** Exercise, mitochondrial dynamics, AMPK

Mitochondrial dynamics is driven by a balance between fusion and fission^[1,2]^. Fusion connects individual mitochondria to form a network of highly energy-efficient organelle systems. Fission drives fragmentation of the mitochondrial network resulting in less energy-efficient organelles. Nevertheless, fission is critical for the removal of damaged mitochondria and the mitigation of oxidative stress. Well-defined factors that control fusion (mitofusin-1/2, optical atrophy protein-1/2) and fission (dynamin-related protein 1, mitochondrial fusion factor, fission protein 1) regulate mitochondrial connectivity dynamics. Mitochondrial dynamics is critical for energy homeostasis, particularly in the skeletal muscle^[1]^, one of the most plastic organs in adulthood amenable to remodeling by exercise, as well as a sedentary lifestyle and aging^[3]^. Exercise has long been known to be necessary for healthy aging and energy efficiency, as well as for delaying cardiovascular and metabolic diseases^[4]^. Exercise benefits are partly mediated by boosting energy metabolism^[1]^. However, molecular mechanisms of age-related decline in physical fitness, and its counter-delay by exercise, are poorly defined. Skeletal muscle is an excellent organ system for obtaining molecular insights into the interaction between exercise and aging, particularly as it is related to mitochondrial dynamics, muscle quality, and fitness. So far, studies in rodents have correlated exercise and fitness with changes in mitochondrial dynamics and quality control^[1]^. However, the direct contribution of mitochondrial processing in skeletal muscle and exercise tolerance during aging, as well as the intervening effect of regular exercise on the age-related decline, remains to be established. Such interactions are difficult to study in larger pre-clinical models such as mice due to the long lifespan in rodents. This warrants studies in model organisms with shorter lifespans and greater amenability to genetic modulation. One such organism is Caenorhabditis elegans (*C. elegans*), which has been widely used to study longevity and aging^[5]^ due to its lifespan of up to 20 days and ease of genetic targeting.

Taking advantage of this model organism in a recent publication^[6]^, Campos *et al*. have characterized the impact of exercise, aging, and anti-aging interventions on mitochondrial dynamics and physical fitness, as well as explored molecular mechanisms that might link physical fitness with mitochondrial dynamics^[6]^. Exercise capacity was first measured in wild-type worms using a swimming assay at different ages (day 1, 5, 10, 15) as a function of body bends, and mitochondrial connectivity (network formation) was measured in body wall muscles. The group found that exercise capacity and mitochondrial connectivity simultaneously decreased with age, such that at day 15, the worms exhibited severely declined exercise fitness and highly fragmented mitochondria. Acute exercise (performed for 1–4 h) resulted in a progressive decline in mitochondrial connectivity, which was restored after 24 h of recovery, indicating exercise-induced fission followed by reparative fusion during recovery. Similar experiments performed at different ages demonstrated an age-dependent decline in exercise fitness, an increase in mitochondrial fragmentation, and poor recovery after 24 h of rest. Physical fitness and exercise recovery inversely corelated with mitochondrial fragmentation and disorganization, and declining mitochondrial plasticity seemed to be responsible for age-related loss in fitness. Indeed, worm orthologs of mitochondrial fusion and fission regulators were critical for physical fitness such that organisms with singular (*fzo-1, drp-1*) or compound (*fzo-1*+*drp-1* and *eat-3*+*drp-1*) mutations in fusion (*fzo-1, eat-3*) and fission (*drp-1*) genes exhibited poor exercise tolerance, training capacity, impaired ability to recover from exercise, and poor mitochondrial connectivity and recovery. A similar phenotype was observed with overexpression of these genes, confirming that physical fitness is governed by plasticity in mitochondrial dynamics rather than fusion or fission alone. Improvement in physical fitness incurred through regular exercise over the lifetime of the worm also required intact mitochondrial fission-fusion dynamics, as worms with defects in fusion genes did not adapt to life-long exercise. Proteomic analysis in wild-type worms revealed that exercise training leads to increased expression of proteins linked to mitochondrial processing, tricarboxylic acid cycle, electron transport chain, oxidative phosphorylation, lipid metabolism and redox balance. Additional pathways upregulated include mitochondrial proteolysis, protein synthesis and calcium handling. Interestingly, exercise training failed to increase these pathways in worms with impaired mitochondrial fusion (*fzo-1* mutants), rather increasing alternatively pathways related to anaerobic glucose metabolism, heat shock response, the ubiquitin-proteasome system, and autophagy. Therefore, intact mitochondrial dynamics seems critical for training-induced aerobic adaptations in the worm muscles.

The authors further explored mechanisms through which exercise training, physical fitness and mitochondrial dynamics are linked. Several anti-aging mutant worms with extended lifespans were measured for physical fitness, exercise recovery, training capacity and mitochondrial connectivity. An array of anti-aging worm lines, including mutants with mild mitochondrial dysfunction (isp-1 and nuo-6), reduced insulin/IGF-1 signaling (rIIS)(daf-2), dietary restriction (DR)-like state (eat-2), and increased AMPK activity (CA-AAK-2), were tested. Surprisingly, most of the longevity pathways, despite extending life span in worms, were dispensable for exercise and mitochondrial capacity and did not prevent the decline in fitness and mitochondrial dynamics with age. AMPK activation was singularly found to be linked to fitness and mitochondrial dynamics. Constitutively active AMPK mutant worms (CA-AAK-2) had greater exercise capacity. However, they lacked the ability to further adapt to exercise training, indicative of an upper limit to exercise tolerance. Conversely, AMPK deficient worms (*aak-2*) not only had poor exercise capacity but also failed at adapting to long-term exercise. Notably, the beneficial effect of constitutively active AMPK on fitness was lost in mitochondrial fusion and/or fission mutant worms (drp-1, fzo-1, drp-1/fzo-1), demonstrating that AMPK activation confers exercise fitness by regulating mitochondrial dynamics. Whether mitochondrial fission-fusion was impaired in loss-of-function AMPK mutants with decreased exercise capacity was not examined.

Campos *et al*. show a clear link between exercise fitness and mitochondrial dynamics in the skeletal muscle and underscore the contributory role of dysfunctional mitochondrial dynamics in declining physical fitness with age^[6]^ [Figure 1]. The authors further identify AMPK as a major orchestrator of muscle function and exercise fitness. The interlinked muscle and mitochondrial aging phenomenon in *C. elegans* unveil new avenues that take advantage of short worm lifespan and powerful genetics to decipher molecular and biochemical signatures of aging in the muscle. For example, the impact of aforementioned interventions on muscle morphology should be measured to determine whether loss of mitochondrial dynamics decreases muscle mass or myofiber size and in turn contributes to age-related loss in muscle function. Recent studies have found a critical role for mitochondrial health in muscle size homeostasis^[7]^, such that mitochondrial dysfunction is associated with muscle wasting in sarcopenia, cachexia, and muscular dystrophy. Whether and how mitochondrial respiration is affected by exercise needs to be characterized in addition to organelle connectivity. While this study identifies AMPK as a central regulator in exercise and muscle aging, the downstream effectors of AMPK remain largely unknown. Genetic screening studies in *C. elegans* could speed up the discovery of other AMPK-dependent regulators of the aging process. Endogenous AMPK signaling in worm muscle walls was shown to be reduced with aging in *C. elegans* but reversed with exercise intervention. In this context, wild-type and mutant worms could be used to screen for novel small molecule activators of AMPK that could be further developed as exercise mimetics for preventing age-related loss of muscle function and physical fitness. Notably, an FDA-approved anti-diabetic drug, metformin, which extends lifespan in worms^[8]^, is an indirect AMPK activator and could affect physical fitness via mitochondrial dynamics. As mentioned above, proteomic adaptation to exercise involving oxidative remodeling was lost in mutant worms with impaired mitochondrial fission compared to wild-type worms. How does change in mitochondrial dynamics trigger muscle oxidative remodeling at the level of the proteome needs further exploration. One speculation is that the changing mitochondrial connectivity and efficiency during exercise training generate distinct metabolites or signaling intermediates that drive oxidative adaptation. Some of the proteomic changes could be driven by underlying transcriptional and epigenetic changes in response to remodeling of the mitochondrial landscape. For example, *C. elegans* orthologs of mammalian oxidative metabolic regulators (e.g., PGC1s and ERRs)^[9]^ could be transcriptional mediators of exercise or AMPK activation. Additional intriguing questions arise: Can the exercise models developed in this study be used to decode whole-body physiological impact via myokines? Can these models help understand the impact of aging on exercise-mitochondria interaction in organs beyond the skeletal muscle? Would novel evolutionarily conserved regulators of fitness and mitochondrial dynamics identified through worm genetics have a broader impact on cardiovascular aging in mammals? Finally, with the emerging role of cellular senescence in aging and cardiovascular disease^[10]^, how is senescence-associated secretory phenotype (SASP) regulated in the reported model system and associated interventions?

In summary, work by Campos *et al*. provides new tools and directions to understand the intertwining impact of aging, exercise, and mitochondrial dynamics on physical fitness, as well as discover molecular drivers of muscle homeostasis that could be translated to exercise mimicking cardiovascular therapeutics for healthy aging^[6]^.

## Figures and Tables

**Figure 1.**
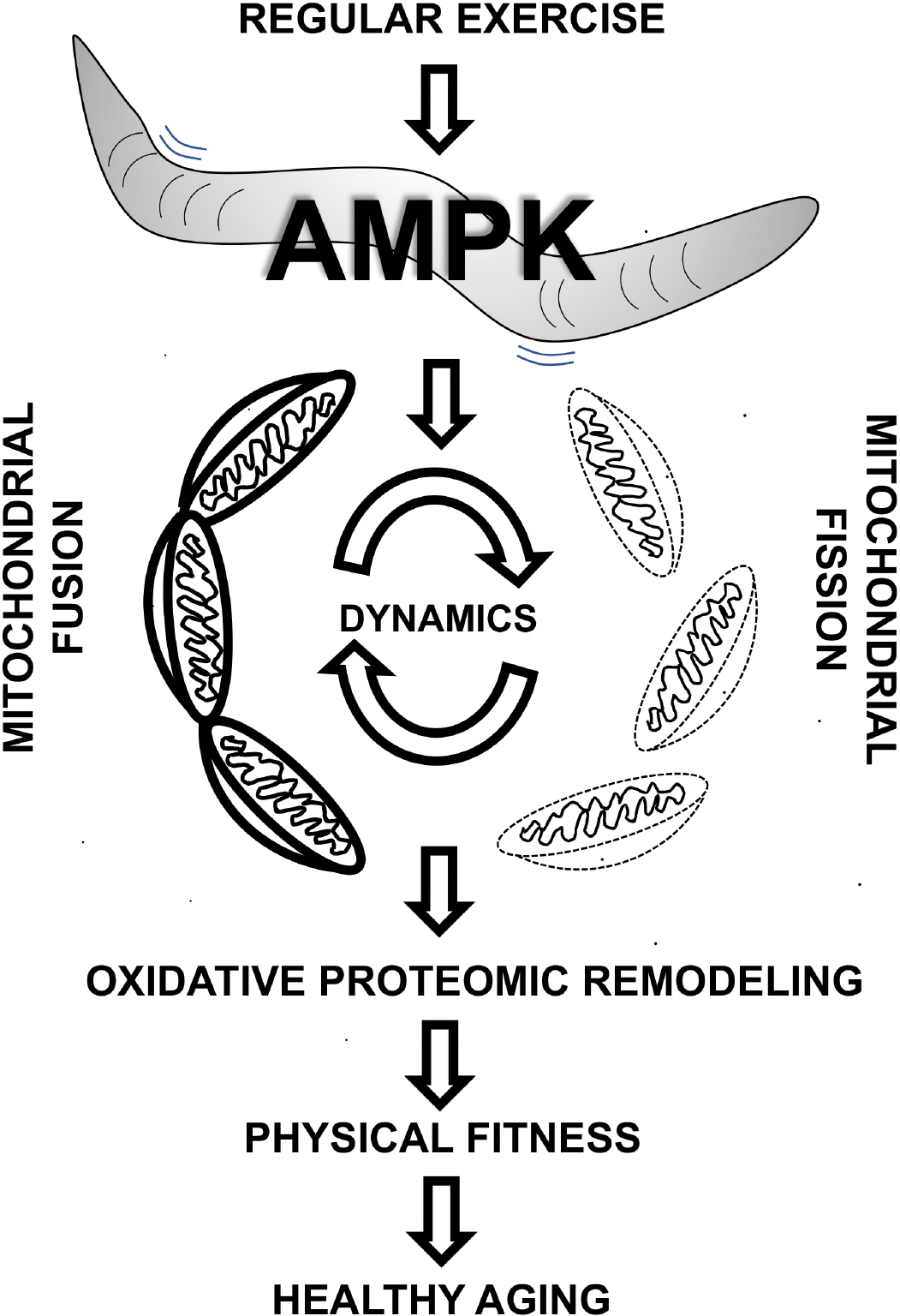
A worm’s life. Evolutionarily conserved AMPK pathway in *C. elegans* is responsible for healthy aging and maintaining physical fitness, by regulating mitochondrial fusion-fission dynamics, and driving oxidative proteomic signature that improves exercise capacity and recovery.
